# The post-orchiectomy systemic inflammatory index is associated with tumor characteristics in clinical stage I germ cell tumors

**DOI:** 10.3389/fonc.2025.1490264

**Published:** 2025-02-24

**Authors:** Peter Lesko, Michal Mego

**Affiliations:** ^1^ 2^nd^ Department of Oncology, Faculty of Medicine, Comenius University and National Cancer Institute, Bratislava, Slovakia; ^2^ Translational Research Unit, Comenius University, National Cancer Institute, Bratislava, Slovakia

**Keywords:** inflammatory, relapse, testicle, stage I, biomarker

## Abstract

**Background:**

Approximately 25% to 30% of clinical stage I (CSI) germ cell cancer (GCT) patients will experience disease relapse after an orchiectomy. Adding adjuvant treatment will decrease the relapse rate but could lead to over-treatment. Prognostic biomarkers such as lymphovascular invasion (LVI) and/or embryonal carcinoma (EC) in non-seminoma (NSGCT) and rete testis invasion (RTI) and/or primary tumor size (PTS) in seminoma (SGCT) add limited value in treatment decision- making. The aim of this study is to assess the systemic inflammatory index (SII) and lactate-dehydrogenase (LDH) with clinicopathological findings along with their prognostic impact.

**Methods:**

This is a retrospective study that included 159 diagnosed CSI GCT patients, who underwent active surveillance (AS) from June 2004 to November 2023. Medical records and pathology reports were collected retrospectively. Drawn blood must have been done less than 3 months after the orchiectomy had been done. For the survival analysis, we used dichotomized values of the studied biomarkers from “low” to “high” based on the median values.

**Results:**

The median follow-up time was 61 months (ranging from 1 to 230 months), with 2-year relapse- free survival (RFS) of 81.3% and 69.0% in SGCT and NSGCT, respectively. We confirm inferior RFS in the presence of LVI compared to an absence of LVI in NSGCT ([HR]= 2.59, 95%CI (0.74-9.07), p=0.04). A trend of inferior RFS in NSGCT patients with EC predominance (≥50%) was also observed ([HR]= 2.59, 95%CI (0.98-6.85), p=0.06). A prognostic impact of RTI and a PTS >4cm in SGCT was not observed with p=0.24 and p=0.51, respectively. The SII was assessed in the population, and a higher neutrophil- to- lymphocyte ratio (NLR) value was associated with LVI presence and with advanced tumor stage in NSGCT. In SGCT, a higher SII level was associated with LVI presence and advanced pathological stage. A PTS >4cm was associated with a higher LDH level among all the studied patients, without significance in SGCT or NSGCT. A higher LDH value in NSGCT was also associated with EC predominance (≥50%).

**Conclusion:**

Our study, for the first time, revealed associations of post-orchiectomy systemic inflammatory indices and/or LDH in CSI GCT. These new associations deserve further evaluation in a larger cohort of patients with CSI GCT to elucidate whether its associations in certain histology subgroups will improve the stratification of the at-risk population.

## Introduction

A testicular germ cell tumor (TGCT) is the most common solid cancer among men < 34 years old (1). Clinical stage I (CSI) testicular germ cell tumors are characterized by a restriction of the tumor to the testicle without any signs of distant spread and postoperative levels of serum tumor markers within the normal range (2). Cure rates for TGCTs are >99% (3, 4). Overall survival (OS) of CSI TCGTs regardless of histological type is approaching 100% independent of the treatment modality; thus, the treatment choice is mostly driven by risks associated with treatment-related toxic effects and by the patients’ quality of life (5, 6). The results from several studies showed that 4% to 50% (7–12) of patients with a TGCT classified as CSI will relapse after an orchiectomy.

Current post-orchiectomy treatment approaches vary between chemotherapy, radiotherapy, and active surveillance (AS) in CSI SGCTs, whereas, CSI non-seminoma germ cell tumors (NSGCTs) lack radiosensitivity (13); thus, treatment approaches for these tumors include AS, adjuvant chemotherapy, and retroperitoneal lymph node dissection (RPLND), according to recommendations by international societies such as NCCN, ESMO, and EAU (14–16).

A current biomarker used in treatment decision- making in CSI NSGCT is the presence of lymphovascular invasion (LVI), however, the use of LVI as a biomarker is accompanied by various pitfalls such as over-treatment in approximately 50% of patients (9, 10). Due to this, many centers do not conduct treatment decision- making based on LVI, thus patients with the presence of LVI are mostly advised to choose active surveillance. In contrast, data regarding a primary tumor size (PTS) >4cm and rete testis invasion (RTI) as biomarkers in CSI SGCT are ambiguous and have led to conflicting results (17, 18), thus treatment decisions based on these biomarkers in CSI SGCT are not justifiable (12, 19). Due to this, there is an effort to investigate new biomarkers for more precise patient stratification. A recent review evaluated biomarkers investigated in stage I TGCT (20).

Blood- based parameters such as neutrophils, lymphocytes, monocytes, platelets, neutrophil- to- lymphocyte ratio (NLR), platelet- to- lymphocyte ratio (PLR), systemic inflammatory index (SII), or lactate dehydrogenase (LDH) and their association with relapse and clinical pathological characteristics were studied in various types of cancer such as colorectal, melanoma, gastric, and breast cancer (21–24). The role of NLR and SII was also evaluated in metastatic TGCTs (25–28), however, its association with clinicopathological features in patients with CSI TGCTs has not been studied yet. It is also known that LDH has a prognostic role and its level is associated with clinical and/or pathological features such as tumor stage, LVI, and node metastasis in solid tumors (29, 30). Moreover, LDH is one of three serum tumor markers that are generally used in assessing clinical staging in germ cell tumors, therefore, providing additional information in treatment decision- making (14–16). However, associations between post-orchiectomy levels with clinical and/or tumor characteristics have not been assessed in precisely selected CSI germ cell tumors yet.

The aim of this study was to assess the relationship between clinicopathological characteristics and systemic inflammatory indexes and/or LDH in patients with CSI TGCTs and to determine their impact on prognosis.

## Materials and methods

### Study design and population

**Figure 1:**
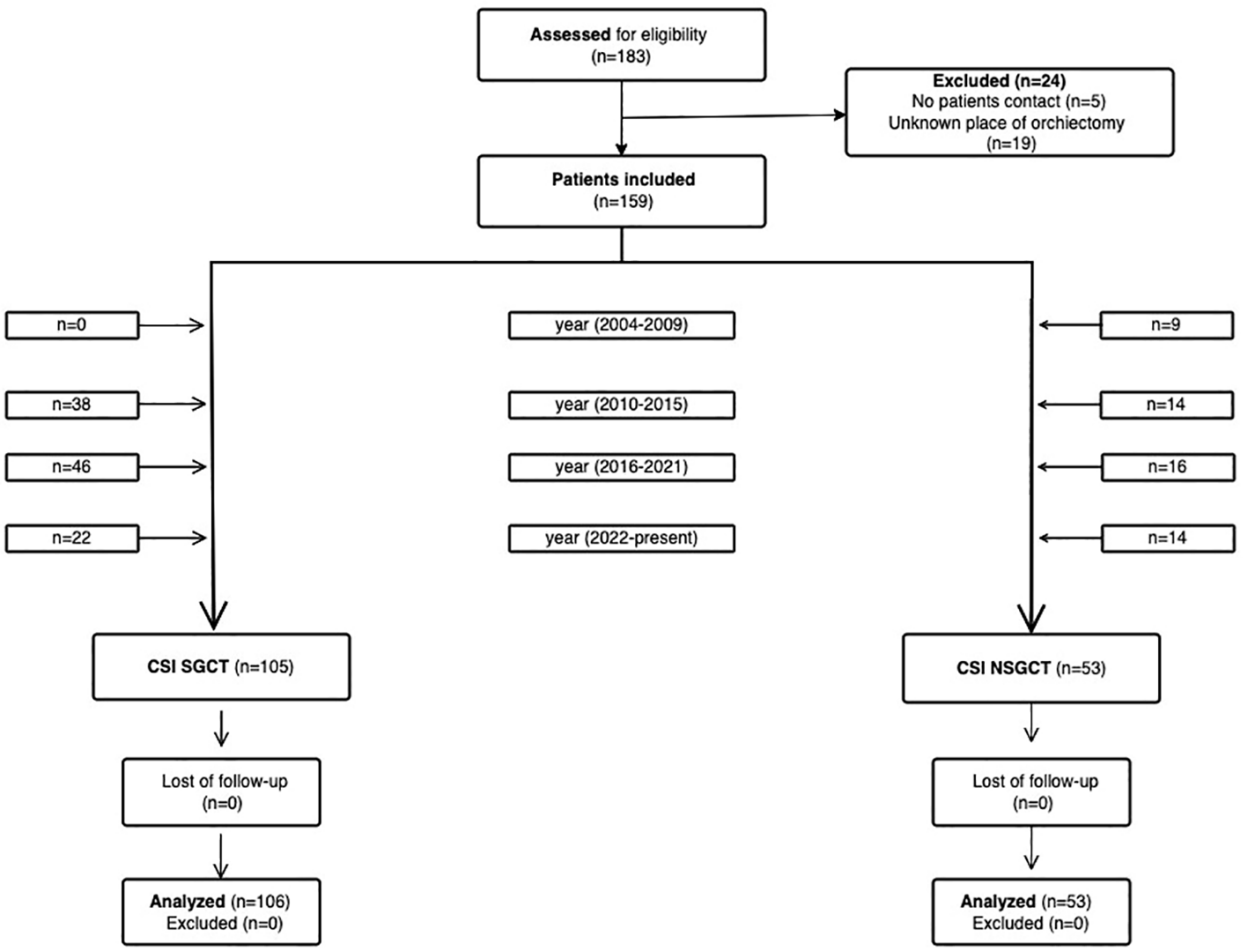
Flow diagram showing patient enrollment from identification to inclusion. No patient contact and data paucity led to patients being excluded. (Created with the Visual Paradigm app).

In our retrospective study, we initially included 183 patients (**Figure 1**). Eligible patients were aged ≥ 18 years old and had a histologically confirmed germ cell tumor that was staged as clinical stage I with drawn blood that must have been done ≤ 3 months after the orchiectomy. Patients for whom the place of orchiectomy was unknown were excluded. Of the initially included patients, 24 patients were excluded due to unknown pathology laboratories or no patient contact in order to find out pathological laboratory. After exclusion, this study included 159 patients with TGCTs, staged as clinical stage I according to ACJJC 8^th^ edition (2) staging. The patients were treated with curative orchiectomy between June 2004 and November 2023 in regional urological departments in the Slovak Republic and most of them underwent active surveillance at the National Cancer Institute (NCI) afterward. Since 2014, the paradigm of treatment has changed towards active surveillance, therefore, most of the patients were included after 2014. Some patients were referred to the NCI due to a relapse being diagnosed. The patients’ blood test results were available in the hospital system. Data regarding tumor histology and other patient/tumor characteristics were collected and correlated with blood draw results and risk of relapse. The study was approved by the institutional review board (IRB project number: IZLO-1) of the National Cancer Institute of Slovakia. Each participant signed informed consent before the study was initiated.

### Laboratory parameter collection

Complete blood count (CBC) and LDH results were collected retrospectively for each study participant from the hospital medical system. The blood draw must have been done less than or equal to 3 months after the orchiectomy had been done. In the case of patients who were referred to our center due to a relapse being diagnosed, laboratory results were requested from the local oncology and/or urology outpatient centers. SII was calculated based on platelet (P), neutrophil (Ne), and lymphocyte (L) counts using a formula described previously (27): SII=P × Ne/L (31). NLR was calculated by the formula: NLR= Ne/L (32) and PLR was calculated by the formula: PLR=P/L (33).

### Pathology examination

Primary TGCTs had been diagnosed in all the included patients. A TGCT was classified according to the current WHO classification (34). Orchiectomy specimens were histo-pathologically examined in pathology laboratories in the Slovak Republic. Pathology reports were requested and collected systematically afterward.

### Statistical analysis

Tumor pathological and clinical data were tabulated. The patients’ characteristics and the CBC and biochemistry parameters were summarized using the mean or median (range) for continuous variables and frequency (percentage) for categorical variables. Statistical analyses were performed using non-parametric tests as the distribution of the neutrophil, lymphocyte, monocytes, platelet, SII, NLR, PLR, and LDH values had a non-normal distribution (Shapiro–Wilk test). The non-parametric Kruskal– Wallis test was used for the analysis of the association between hematological and biochemistry parameters and clinicopathological variables in the two groups of patients. The independence of the variables in the two groups with dichotomized values for each parameter (“low” vs “high” based on the median value) was compared using Fisher’s test. Relapse-free survival (RFS) was calculated from the date the orchiectomy was performed to the date of progression or the date of the last adequate follow-up. OS was calculated from the date the orchiectomy was performed to the date of death or last follow-up. The univariate Kaplan–Meier statistical approach was used to assess the outcome of survival data in certain populations in our studied groups. All p- values presented are two-sided, and associations were considered significant if the p- value was less than or equal to 0.05. Statistical analyses were performed using NCSS 2022 statistical software.

## Results

### Patients characteristics

From June 2004 to November 2023, this study encompassed 159 patients with diagnosed TGCTs with a median follow-up of 61 months (ranging from 1 to 230 months). **Table 1** summarizes the patient and tumor characteristics. The majority of the patients were classified as seminoma and pathologically staged as pT1. Relapses were observed in 41 patients (25.8%), with a median time to relapse of 9 months and a median follow-up of 111 months. In CSI NSGCT, relapses occurred in 18 (33.96%) patients with a median time to relapse of 5.5 months and a median follow-up of 72 months, whereas in the CSI SGCT subgroup, relapses were observed in 23 (21.70)% patients with a median time to relapse of 11 months and a median follow-up of 56 months. RFS at 2 years was 81.25% and 69.04% for the CSI SGCT and NSGCT subgroups, respectively.

**Table 1:** Patients’ characteristics.

Patients’ characteristics	All patients N (%)	CSI SGCT N (%)	CSI NSGCT N (%)
All patients	159 (100.0)	106 (66.7)	53 (33.3)
Size of tumor			
>4cm	56 (35.2)	40 (25.6)	16 (10.3)
≤4cm	100 (62.9)	64 (41.0)	36 (23.1)
NA	3 (1.9)	–	–
Relapse			
Relapse	41 (25.8)	23 (14.5)	18 (11.3)
Without relapse	118 (74.2)	83 (52.2)	35 (22.0)
Relapse period			
Early relapse (<24 months)	36 (22.6)	20 (48.8)	16 (39.0)
Late relapse (≥24 months)	5 (3.1)	3 (7.3)	2 (4.9)
Tumor characteristics			
pT1	109 (68.6)	69 (43.4)	40 (25.2)
pT2	46 (28.9)	35 (22.0)	11 (6.9)
>pT2	4 (2.5)	2 (1.3)	2 (1.3)
Rete testis invasion			
Present	57 (35.9)	44 (22.7)	13 (8.2)
Absent	87 (54.7)	58 (36.5)	29 (18.2)
NA	15 (9.4)	–	–
Embryonal carcinoma predominance (≥50%)			
EC predominance present	–	–	22 (13.8)
EC predominance absent	–	–	31 (19.5)
LVI			
LVI present	48 (30.2)	37 (23.3)	11 (6.9)
LVI absent	111 (69.8)	69 (43.4)	42 (26.4)
Teratoma in primary			
Teratoma present	–	–	31 (19.5)
Teratoma absent	–	–	22 (13.8)
NSGCT type			
Pure embryonal carcinoma	–	–	4 (7.5)
Pure teratoma	–	–	1 (1.9)
Mixed TGCT	–	–	48 (90.6)

TGCT, testicular germ cell tumor; NSGCT, non- seminoma germ cell tumor; SGCT, seminoma germ cell tumor; CSI, clinical stage I; pT, pathological stage; LVI, lympho vascular invasion; EC, embryonal carcinoma; N, number of patients; NA, not available.

Late relapses (≥24 months) were detected in five patients (3.1%), of whom four4 patients were classified as CSI SGCT and one as CSI NSGCT. Their median time to late relapse was 38 months with a median follow-up of 89 months.

### Prognostic association of tumor and patient’s characteristics with RFS

We investigated the prognostic association between tumor and/or patients’ characteristics and RFS in all patients and in subgroup analyses in seminoma and non-seminoma histologies separately.

In the seminoma subgroup analysis, we were not able to prove any prognostic association between tumor and/or clinical characteristics and RFS. Neither rete testis invasion [hazard ratio (HR) = 0.60, 95% CI (0.27-1.37), p=0.240] nor PTS >4cm [HR = 1.12, 95% CI (0.492.58), p=0.781] were associated with survival outcomes.

**Figure 2:**
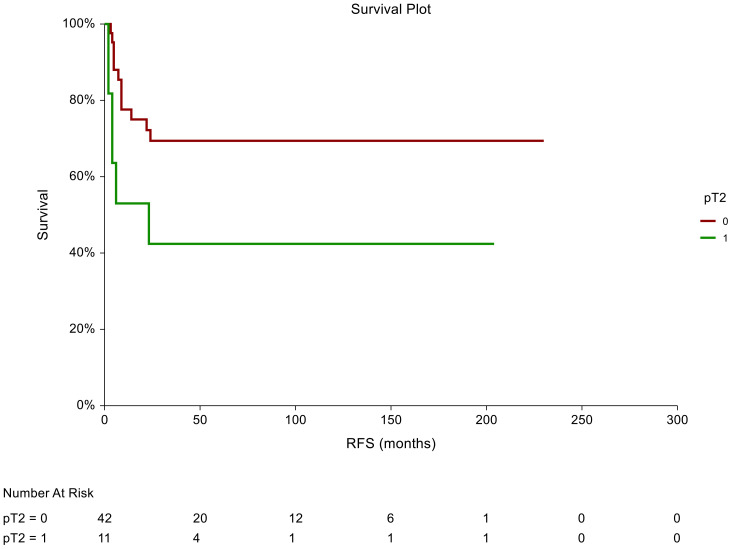
Kaplan–Meier estimates of probabilities of relapse-free survival according to pT2 tumor characteristics in patients with TGCTs (N = 159). Patients with non-pT2 tumor histology had significantly better RFS compared to patients with pT2 [HR= 2.72, 95%CI (0.76-9.072, p=0.034)]; 0 = non- pT2 tumor stage in tumor specimen, 1 = pT2 tumor stage in tumor specimens. HR, hazard ratio; TCGT, testicular germ cell tumor; RFS, relapse- free survival.

**Figure 3:**
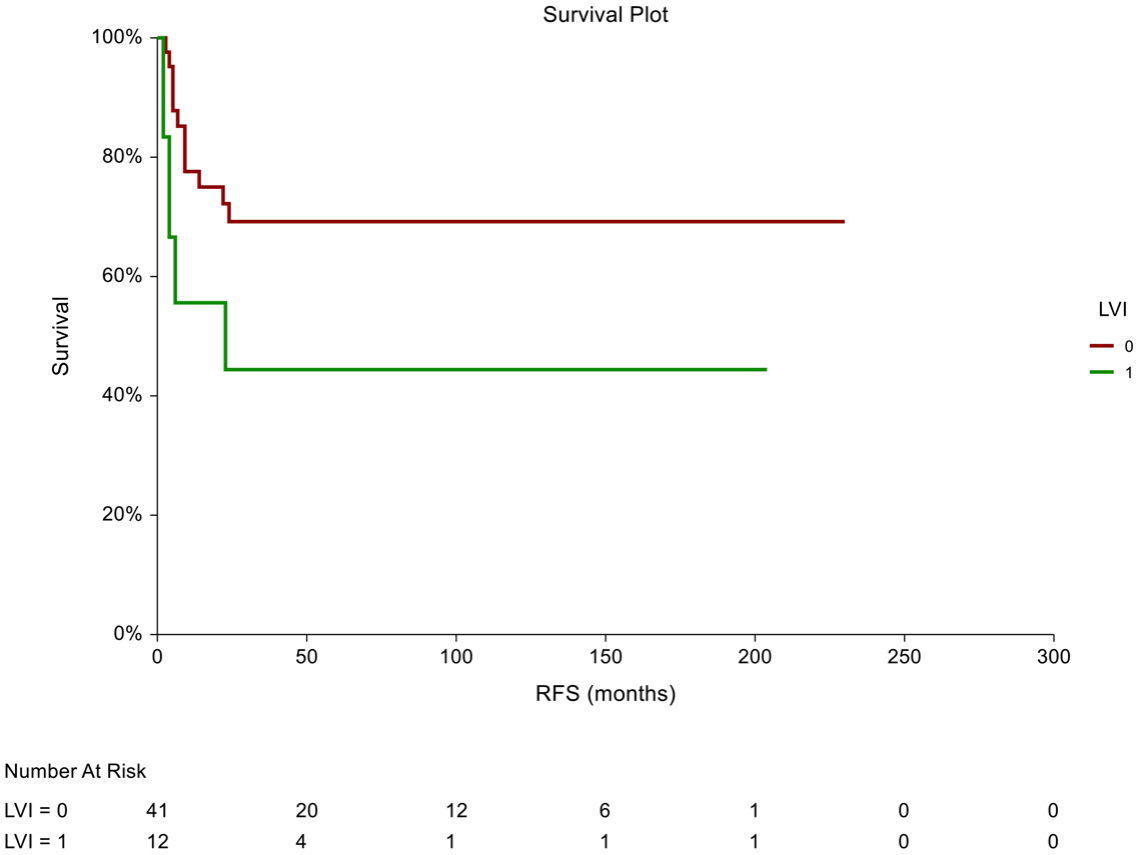
Kaplan–Meier estimates of probabilities of relapse-free survival according to LVI tumor characteristics in patients with TGCTs (N = 159). Patients with the absence of LVI had significantly better RFS compared to patients with LVI [HR= 2.59, 95%CI (0.74-9.07), p=0.044]; 0 = LVI absent, 1 = LVI presence in tumor histology specimens. HR, hazard ratio; TCGT, testicular germ cell tumor; RFS, relapse- free survival.

In the non-seminoma subgroup analysis, the prognostic significance of certain tumor characteristics was retained in patients with LVI presence [HR = 2.59, 95% CI (0.74-9.07), p=0.044 for RFS] (**Figure 2**). Similarly, we observed prognostic significance in tumors that were pathologically staged as pT2 [HR = 2.72, 95% CI (0.76-9.072, p=0.034 for RFS)] (**Figure 3**). EC predominance (50%) was not significantly associated with inferior RFS, however, a trend of inferior RFS in CSI NSGCT patients was observed, [HR= 2.59, 95% CI (0.98-6.85), p=0.062].

In all patients, there was a statistically significant prognostic association between RFS and tumors pathologically staged as pT2 [HR = 1.9, 95%CI (0.94-3.85), p=0.039]. Moreover, we observed an association between RFS and lymphovascular invasion as patients who were LVI+ in the pathological evaluation had a poorer RFS than those who were LVI- [HR = 1.87, 95%CI (0.92-3.76), p=0.046].

### Association between investigated parameters and tumor/patient characteristics

The mean value of post-orchiectomy neutrophils, platelets, lymphocytes, monocytes, SII, NLR, PLR, and LDH ± SEM were compared with the pathological variables of the tumor specimens and the clinical variables in all the patients (**Table 2**).

**Table 2:** Associations between investigated biomarkers (neutrophils, platelets, lymphocytes monocytes, SII, NLR, PLR, and LDH) and clinical and/or tumor characteristics.

Investigated biomarker		All patients (p -value)	CSI SCGT (p- value)	CSI NSGCT (p- value)
Relapse	Positive correlation	–	–	–
	Negative correlation	–	–	–
pT1	Positive correlation	NLR (p=0.011)	SII (p=0.023)	NLR (p=0.053)
	Negative correlation	–	–	–
PTS >4cm	Positive correlation	LDH (p=0.031)	–	–
	Negative correlation	–	–	–
pT2	Positive correlation	SII (p=0.054), NLR (p=0.029)	SII (p=0.042)	NLR (p=0.039)
	Negative correlation	–	–	–
LVI	Positive correlation	SII (p=0.054), NLR (p=0.007)	SII (p=0.023)	NLR (p=0.028)
	Negative correlation	–	–	–
EC predominance	Positive correlation	–	–	LDH (p=0.027)
	Negative correlation	–	–	–
Teratoma	Positive correlation	–	–	–
	Negative correlation	–	–	–
RTI	Positive correlation	–	–	–
	Negative correlation	–	–	–

TGCT, testicular germ cell tumor; NSGCT, non- seminoma germ cell tumor; SGCT, seminoma germ cell tumor; CSI, clinical stage I; pT, pathological stage; LVI, lympho vascular invasion; EC, embryonal carcinoma; RTI, rete testis invasion; PTS, primary tumor size; Neu, neutrophils; Plt, platelets; Lymph, lymphocytes; Mono, monocytes; SII, systemic inflammatory index; PLR, platelet- to- lymphocyte ratio; NLR, neutrophil- to- lymphocyte ratio; LDH, lactate dehydrogenase; EC, embryonal carcinoma predominance ≥ 50%; Teratoma, any percentage of teratoma in histology.

We did not observe any significant associations between neutrophils, platelets, lymphocytes, monocytes, SII, NLR, PLR, or LDH value and relapse in the histological subgroups for seminoma and non-seminoma separately or in all the patients. We were not able to prove any association between LDH value and relapse in the subgroups (seminoma and non-seminoma) or in all patients.

In the seminoma subgroup, there was a significant correlation between SII value and pT2 tumor stage, as patients with a higher SII level tended to be staged as pT2 (618.3 ± 73.21 vs 495.76 ± 51.40, p=0.042). In contrast, our results showed an association between pT1 tumor stage and SII index, where patients with a CSI SGCT with a lower SII level tended to have a tumor staged as pT1 (491.84 ± 72.08 vs 622.55 ± 72.08, p=0.023). An association between NLR and tumor stage in patients with CSI SGCTs was not noted in the pT1 or pT2 population with p=0.113 and p=0.201, respectively. In the non-seminoma subgroup of patients, we observed that pathological tumor staging correlated with NLR count, thus, a lower NLR count was associated with pT1 tumor stage (2.13 ± 0.17 vs 2.53 ± 0.30, p=0.053) and, in contrast, a higher NLR count was associated with pT2 pathological tumor staging (2.44 ± 0.33 vs 2.18 ± 0.17, p=0.039). However, an association between tumor stage and SII index in patients with CSI NSGCTs was not observed with p=0.68. The patients in the unselected cohort of patients with CSI TGCTs with lower NLR values were more prone to be pathologically staged as pT1 (2.22 ± 0.13 vs 2.60 ± 1.93, p= 0.011), which was confirmed by a further association between pT2 pathology staging and higher NLR count (2.53 ± 0.20 vs 2.25 ± 0.13, p= 0.029). Moreover, a borderline positive association between SII and tumor stage in all the patients was observed as patients staged as pT2 were associated with higher SII values (588.85 ± 56.4 vs 501.58 ± 36, p= 0.054).

Our study proved an association between tumor size (PTS >4cm) and LDH post-orchiectomy level in the unselected CSI TGCT population. A primary tumor size >4cm was positively associated with LDH value, thus, a higher LDH level was associated with larger tumors (PTS >4cm) (2.95 ± 0.09 vs 2.73 ± 0.06, p= 0.018). This statistical significance was not retained in the patients with CSI NSGCTs or CSI SGCTs with p=.258 and p=0.066, respectively. However, a trend towards statistical significance was observed in the patients with CSI SGCTs.

**Figure 4:**
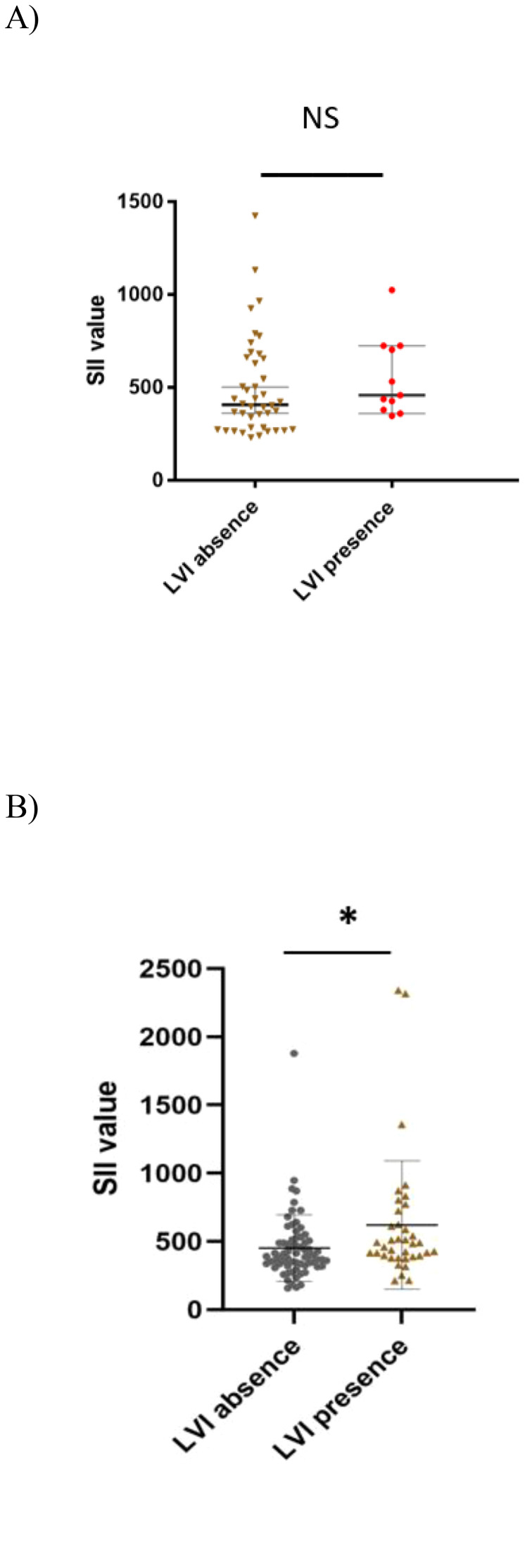
Association between systemic inflammatory index and lymphovascular invasion in CSI NSGCT and CSI SGCT. **(A)** CSI NSGCT. NS, non- significant p- value (> 0.05); SII, systemic inflammatory index; LVI, lympho vascular invasion. **(B)** CSI SGCT. *, p- value < 0.05; SII, systemic inflammatory index; LVI, lympho vascular invasion.

**Figure 5:**
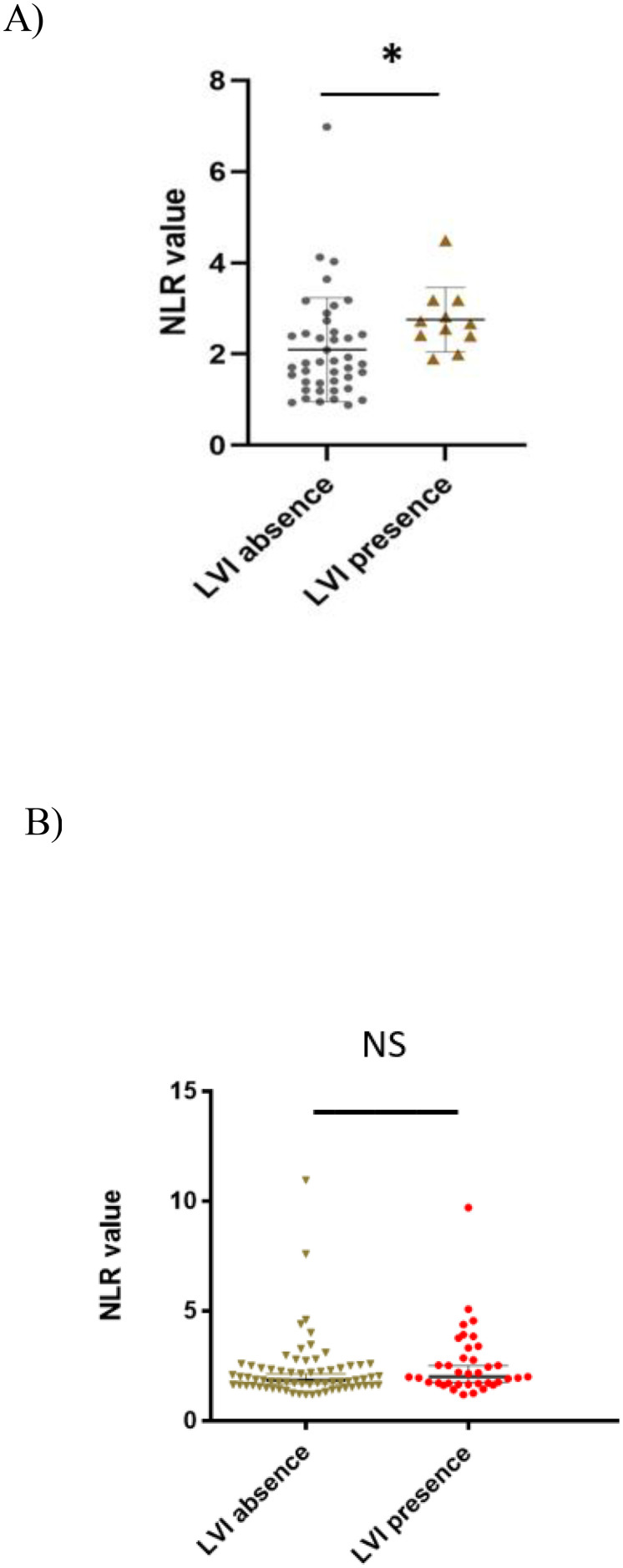
Association between neutrophil- to- lymphocyte ratio and lymphovascular invasion in CSI NSGCT and CSI SGCT. **(A)** CSI NSGCT. *, p value < 0.05; NLR, neutrophil- to- lymphocyte ratio; LVI, lympho vascular invasion. **(B)** CSI SGCT. NS, non-significant p- value (> 0.05); NLR, neutrophil- to- lymphocyte ratio; LVI, lympho vascular invasion.

Additionally, our study found an association in the seminoma subgroup analysis, where we confirmed a significant association between SII and LVI presence (622.55 ± 72.09 vs 491.84 ± 51.70, p= 0.023). However, this was without statistical significance in the selected CSI NSGCT population with p=0.318 (**Figure 4**). This association was also retained in the unselected cohort of CSI TGCT. Thus, in the unselected cohort of patients with CSI TGCTs a higher SII value tended to be associated with being LVI + (601.72 ± 55.0 vs 494.44 ± 36.0, p=0.017). LVI presence in the patients with CSI NSGCTs was positively associated with NLR value (2.61 ± 0.31 vs 2.12 ± 0.16, p=0.039) but this significant association was not retained in the patients with CSI SGCT with p=0.113 (**Figure 5**).

**Figure 6:**
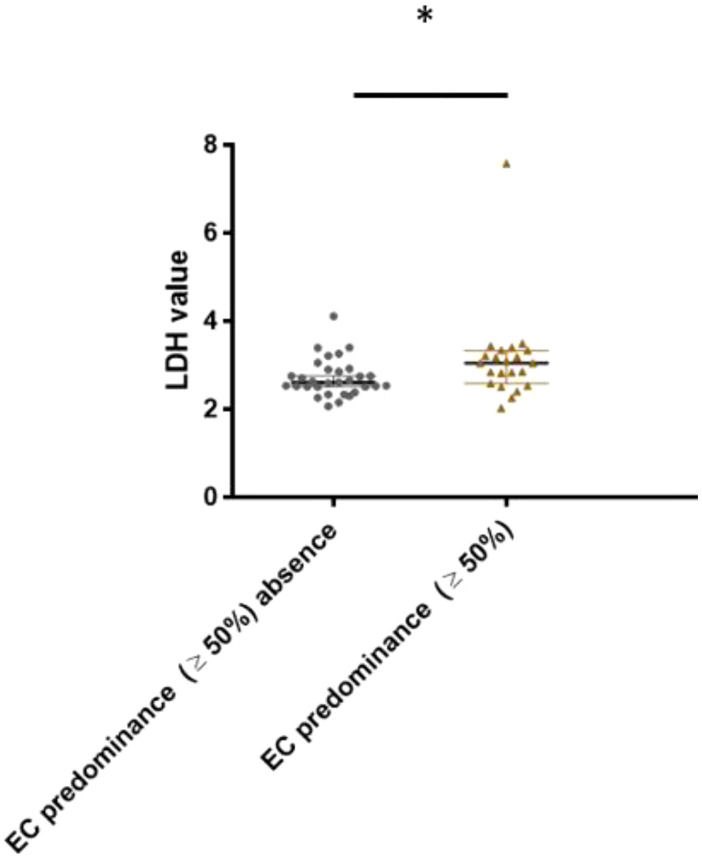
Association between lactate dehydrogenase level and embryonal carcinoma predominance (cut- off ≥ 50%) in CSI NSGCT. *, p value < 0.05; EC, embryonal carcinoma; LDH, lactate dehydrogenase.

This study also proved an association between EC predominance ( ≥50%) and CSI NSGCT histology (**Figure 6**). Patients with a higher LDH level tended to have EC predominance in their histology tumor specimens (3.13 ± 0.16 vs 2.71 ± 0.14, p=0.027).

## Discussion

In this retrospective study, we confirmed the prognostic role of LVI+ and pT2 in patients with CSI NSGCTs. The prognostic impact was significant in all the studied patients. However, these associations were not observed in the CSI SGCT subanalysis. The explanation is that the significance in all patients was derived from the inclusion of patients with CSI NSGCTs. In the CSI SGCT histology subgroup, we did not confirm the prognostic impact of a PTS of >4cm and RTI, therefore, our data added inconsistent results to the ambiguity (35–38) of prognostic biomarkers in CSI SGCT, thus the clinical utility of these biomarkers is not justifiable.

Several studies have proposed that LVI presence is associated with 2.2 to 3.2 times poorer relapse- free survival than the absence of LVI (39, 40). These data are consistent with our results, as almost 2.6 times poorer relapse- free survival compared to LVI- patients was observed. Our results also showed the prognostic impact of pT2 on RFS among all the studied patients, as patients with CSI NSGCTs staged as pT2 tended to have approximately 2.7 times poorer survival compared to the non-pT2 patients. The reason for our significant association might be explained by the high number of LVI + patients in pT2 pathologically staged patients, which was 100% of the patients. Studies evaluating tumor stage as a risk biomarker have shown conflicting results (41–43). Unfortunately, in our study, we were not able to prove any significant prognostic association between RFS in CSI NSGCT and EC predominance in tumor histology representation with p=0.062. Despite this association not being statistically significant, there was a trend of poorer relapse-free survival in patients who presented with EC ≥50%. A recent meta-analysis (44) found that EC predominance in histology was associated with a higher odds of experiencing occult metastasis (OR 2.62, 95% CI 1.93–3.56; p< 0.001) (44). Therefore, the statistical insignificance in this study could be caused by the small sample size with EC predominance.

To the best of our knowledge, NLR in patients with TGCTs has been studied in pre-treatment and post-treatment (25, 26, 45, 46),however, a study focused on relapse in patients with CSI TGCTs has not yet been conducted. Pre-orchiectomy NLR was studied in a cohort of 130 unselected patients with TGCTs who underwent radical orchiectomy. In this study, NLR >4 was associated with >pT1 tumor stage (45). Another study (25) assessed pre-orchiectomy NLR in 160 patients with TGCTs, of whom more than 60% were staged as CSI. Lymph node involvement was associated with pre-orchiectomy NLR ≥3.0 with an odds ratio (OR) of 2.91 (95% CI, 1.67–5.83; p=0.038) (25). Another study evaluated NLR post-orchiectomy (26). This study included 80 patients, of whom 40% were staged as CSI. In this study, NLR was independently associated with relapse in patients with CSI TGCTs with an HR of 1.85 (CI 95% 0,99—3,46), however, the p- value was not stated. The study also proposed that a post-orchiectomy NLR of >2.255 is a predictor for recurrence with an area under the curve of 78.7% (26). However, only 16 recurrences occurred in the studied cohort (including stage I and stage II), therefore, the results from this study are only hypothesis- generating. Our results addressed similar correlations between NLR and tumor histology staging, where pT1 was associated with lower NLR value than patients with ≥pT2 in all patients (p=0.011) and the NSGCT subgroup (p=0.053). LVI was also significantly associated with a higher NLR value compared with LVI- negative patients in the non-seminoma histology subgroup of patients. Given that, in previous studies, the NLR cut- off value for prediction of recurrence was > 2.255, our mean and median value was 2.61. Despite the higher NLR in our study, we were not able to prove concordance with a previous study (26). This could be due to the fact that our cohort of patients was more focused on CSI and our cohort consisted of >40 relapses.

PLR is another marker of systemic inflammationthat has been studied as a prognostic parameter in various cancers such as ovarian and gastric cancer and hepatocellular carcinoma (47). A study addressing the significance of PLR in TGCT proposed that there was a difference in PLR value observed between healthy donors and patients with GCT with p<0.05 (48). However, its prognostic role in TGCT has not been studied. Therefore, this is the first study that has assessed the association between post-orchiectomy PLR value and clinical and/or pathological TGCT characteristics. Nevertheless, we were not able to prove a prognostic impact of PLR on PFS nor were we able to prove any association with other investigated clinical and/or pathological characteristics.

It is known that SII reflects the local or systemic inflammatory response, which is known to have a prognostic association with PFS and/or OS and SII in cancers such as colorectal, pancreatic, and stomach cancer (28, 49, 50). The prognostic role of SII has already been investigated among patients with TGCTs treated with conventional and/or high- dose chemotherapy (27, 51), where it was shown that SII was inversely correlated with PFS and OS (27, 51). The authors in a study (27) proposed a cut- off value of SII≥1003 for shortened PFS and OS, however, this study only included 5% of patients with non-metastatic TGCTs. Another study (52) included approximately 60% of patients with CSI TGCTs and pre-orchiectomy SII was studied. There was no association found between pre-orchiectomy SII and LVI, pathology staging, or tumor size (52). A recent study evaluated SII in pre-orchiectomy manner, where approximately 50% of the patients in the study were staged as CSI. The study found that an SII <881.24 was associated with CSI compared to an SII ≥881.24 and an association with ≥CSII was noted (53). Therefore, extrapolating associations in patients with CSI remains unclear due to different study designs and different populations. In summary, SII in a precisely selected stage I TGCT population has not been recently studied. Surprisingly, in our study, we found an association between SII and pT2 tumor stage and LVI+ in all patients with p=0.054 and p=0.016, respectively. Moreover, tumor stage in patients with CSI SGCTs was also positively correlated with SII value. Regardless of these findings, we were not able to prove any prognostic association or additional association with other clinical/pathological features such as EC predominance, RTI, or teratoma presence. The reason why we did not observe any prognostic impact of SII could be explained by the highly selected population without systemic dissemination and with a minimum of patients who could have had occult disease at the time of their blood being drawn. Moreover, the mean SII value in the patients without relapse in our study regardless of histology subtype was <536, therefore, according to the cut- off values proposed by other studies (27, 28), this may explain why the prognostic impact was not pronounced. However, the predefined cut- off values were proposed for drawing blood pre-orchiectomy blood drawn. The difference in LVI + patients could be attributed to the fact that vascular invasion leads to more imminent attraction of inflammation cells into the tumor surroundings, thus, leading to a change in SII value, which could mirror tumor microenvironment changes. Our hypothesis that tumor microenvironment changes evaluated by SII was associated with relapse was not observed, however, an association with lymphovascular invasion was observed. The proposed associations may add an additional role for SII in more precise stratification of at-risk patients.

Elevated levels of serum LDH are always observed in cancer due to tissue injury following the release of LDH into serum upon cell death (54). LDH is also one of three markers generally used in assessing clinical staging in TGCT, thus adding additional information for treatment decision- making (14–16). However, LDH seems to have minimal impact in assessing recurrence as a solitary marker on AS and its level is more prominent in the advanced stage of disease (55). In concordance with previous studies, (14–16, 55) the LDH level in our studied cohort was below the upper normal value (≤4.1ukat/L) and there was no association between relapse and post-orchiectomy LDH level with p=0.518. Surprisingly, in all the patients, we found that LDH was positively associated with a tumor >4cm with p=0.018. Moreover, in the CSI NSGCT subgroup, we found that EC predominance was associated with higher LDH levels with p=0.027. It has been found that the half-life of LDH is 24 hours (56), however, a study that evaluated the dynamics of LDH in stage I TGCT proposed that the decay of serum LDH after orchiectomy could take 1 to 3 weeks but depends on the initial LDH value (57). This could be an explanation for the positive association between LDH and tumor volume, even with the blood being drawn after the orchiectomy, due to higher values during its decay. Moreover, our study found an association with EC predominance, where EC predominance in the tumor histology specimen was positively associated with LDH value. This could be explained by more aggressive forms of TGCT with aggressive behavior and rapid cell turnover (58) that most probably caused elevated LDH levels, which could have been eliminated after the orchiectomy.

The reason why we observed significant correlations between NLR and the aforementioned biomarkers including LVI, pT2, and pT1 could be explained by the fact that neutrophils are a marker of systemic or local inflammation (59), which could have tumor- promoting and tumor- suppressing effects (60). Furthermore, lymphocytes are part of the adaptive immune system response and could be ameliorated by neutrophils (61). Therefore, neutrophilia with lymphocytopenia represents a significant decline in cell adaptive response (61). Neutrophils play a role in promoting cancer and metastasis, which is mostly associated with neutrophils in the tumor micro-environment called tumor- associated neutrophils (TAN). TAN can be polarized into the N1 population, which has an anti-tumor effect, and N2, which has a pro-tumorigenic effect (62). N2 neutrophils encompass granules with high levels of matrix metalloproteinase-9 (MMP-9) and arginase 1 (ARG-1) (63). MMP-9 degrades the extracellular matrix and releases VEGF to promote angiogenesis along with cancer cell intravasation. (64, 65) Moreover, the release of AGR-1 has a significant effect, causing the inhibition of CD-3 mediated T cell activation and proliferation, thus promoting immunosuppression (66). Moreover, neutrophils secrete a spectrum of cytokines such as interleukin-1B, TNF-a, interleukin-6 (IL-6), and IL12 (61), which induce a chronic inflammatory process and are known to promote angiogenesis, and thus, tumor progression (67). Neutrophilia is always a sign of advanced cancers. It has been hypothesized that tumor production of granulocyte-macrophage colony-stimulating factor (GM-CSF) could be responsible for the increased value of neutrophils (68). Another study proposed the hypothesis that granulocyte- colony-stimulating factor (G-CSF), IL-1, and IL-6 production by tumors may also contribute to higher neutrophil levels (69). Therefore, neutrophils may enhance tumor progression, thus causing an increase in neutrophil values.

SII is an inflammation parameter that consists of NLR multiplied by the level of platelets (31). The association between SII and LVI and/or pT2 tumor staging could be explained by a few hypotheses. Platelets play a role in cancer development from initiation to metastasis. The association with LVI could arise from the fact that platelets are one of many spectrum cells incorporated in the tumor microenvironment (70). Platelets are usually present in abundant tumor vessels where they secrete angiogenic factors such as stromal cell- derived factor, basic fibroblast factor, and VEGF, thus promoting angiogenesis (71). Moreover, the number of platelets changes during cancer development. Patients with active cancer have higher levels of G-CSF and IL-6 (72), which results in higher platelet counts (73). Therefore, platelets are part of the systemic inflammatory response, which may be elevated in cancer, however, their solitary role in GCT in our study was without any associations.

To the best of our knowledge, precise prognostic biomarkers in the assessment of relapse in CSI TCGT are still missing. Several biomarkers have been prognostically investigated in CSI TCGT, however, none of them have been clinically applied, except LVI, but it has drawbacks (20). The advantage of our studied biomarkers is that they are easy to implement and require cost-effective examinations that can be conducted in almost every out-patient and in-patient healthcare provider. On the other hand, the most promising biomarker in TGCT is miRNA 371a-3p. MiRNA 371a-3p is currently being investigated as a diagnostic tool to assess recurrence in stage I TCGT (74). It is known that post-orchiectomy miRNA value and/or percentage of decline between pre- and post-orchiectomy is not prognostic in CSI (75), however, using miRNA as a diagnostic biomarker in relapse could yield a diagnosis of relapse in CSI TGCT sooner (76). Despite this, several major drawbacks need to be resolved before the use of miRNA371a-3p in clinical practice, such as the standardization of technique, cut-off values for diagnosis, and the therapeutic approach in the case of elevated miRNA371a-3p levels post-orchiectomy (77). Moreover, stage I TCGT is a unique oncological entity, where the oncological therapeutic approach is mostly driven by toxicity due to similar OS regardless of the treatment approach (5, 6).Therefore, implementing miRNA 371a-3p in clinical practice would need to diminish false positive results despite its high sensitivity and specificity, and the impact of a biomarker- driven treatment approach needs to further validated (76).

This study has several limitations. One of them is the time of follow-up. It is known that > 95% of relapses occur in <2 years after an orchiectomy. However, 25% of the patients in our cohort consisted of patients who were observed less than 2 years after their orchiectomy. Therefore, potential relapses could be experienced in the future, which may contribute to data uncertainty. Another study limitation is that our cohort consisted mostly of patients with SGCTs, with 66%, compared to 34% of patients with NSGCTs. Moreover, discrepancies between pathology laboratories were present as well. Some pathology laboratories did not assess RTI or tumor size or provided unclear histological proportions in certain specimens. Therefore, central laboratory reassessment would add additional certainty to the pathology reports, which may enhance our data results and may provide more significant associations. In several cases, we needed to contact regional oncology outpatient clinics to find out post-orchiectomy blood drawing results. Thus, in some cases, blood samplings and laboratory methods and their standardization may vary. Furthermore, our study included 159 patients, in whom 41 relapses occurred, of which 18 were classified as non-seminoma and 23 as seminoma. Therefore, the size of the cohort in our study is appropriate for hypothesis- generating results. The advantage of our study is that it focused on stage I GCT, where we assessed the association between blood-based biomarkers and relapse and other clinical and/or tumor characteristics. To the best of our knowledge, these associations have not been studied in this highly selected population.

In conclusion, this is the first report that revealed associations between post-orchiectomy hematological parameters and/or LDH in CSI TGCT. These new associations deserve further evaluation on a larger cohort of CSI TGCT to elucidate whether the associations in certain histological subgroups will improve the stratification of the at-risk population.

## Data Availability

The raw data supporting the conclusions of this article will be made available by the authors, without undue reservation.
